# Poly[aqua­bis­[μ_3_-4-(3-pyrid­yl)pyrimidine-2-sulfonato-κ^4^ *N* ^4^:*N* ^1^,*O*:*O*][μ_2_-4-(3-pyrid­yl)pyrimidine-2-sulfonato-κ^3^ *N* ^4^:*N* ^1^,*O*]tris­ilver(I)]

**DOI:** 10.1107/S1600536811026626

**Published:** 2011-07-09

**Authors:** Xia Liu, Dahua Hu

**Affiliations:** a Department of City Science, Jiangsu City Vocation College, Nanjing 210003, People’s Republic of China

## Abstract

In the crystal structure of the title compound, [Ag_3_(C_9_H_6_N_3_O_3_S)_3_(H_2_O)_2_]_*n*_, the mol­ecules are linked into three-decked polymeric zigzag chains propagating in [100]. On the middle deck, the Ag atom is five-coordinated by three O atoms from three 4-(3-pyrid­yl)pyrimidine-2-sulfonate (*L*) ligands, one of which lies on a mirror plane with the sulfonate group disordered over two orientations in a 1:1 ratio, and two N atoms from two *L* ligands, which lie on the same mirror plane. On the upper and lower decks, the Ag atom is four-coordinated by an aqua ligand, one O and two N atoms from two *L* ligands with the pyridyl and pyrimidine rings twisted at 19.8 (2)°. In the polymeric chain, there are π–π inter­actions between six-membered rings of *L* ligands from different decks with centroid–centroid distances of 3.621 (7) and 3.721 (3) Å. In the crystal, inter­molecular O—H⋯O hydrogen bonds link further these three-decked chains into layers parallel to (010).

## Related literature

For backgroud to coordination polymers with thio­ethers, see: Dong *et al.* (2009 ▶); Fang *et al.* (2010 ▶). For the crystal structure of the related compound *catena*-poly[[μ-4-(2-pyrid­yl)-pyrim­idine-2-sulfonato)-silver(I)] monohydrate], see: Zhu (2010 ▶).
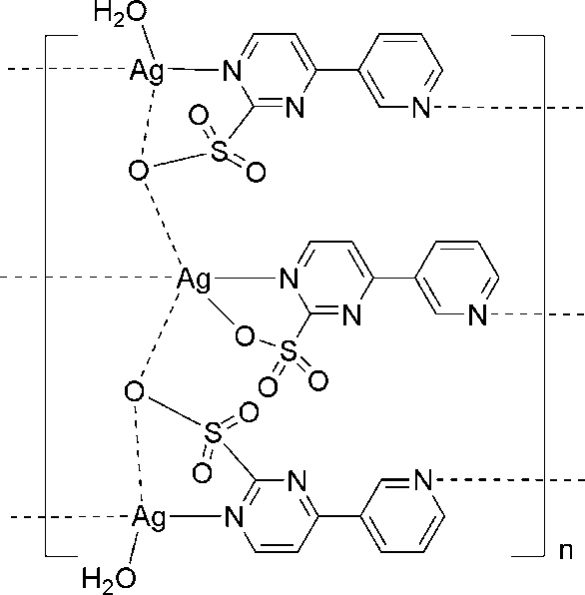


## Experimental

### 

#### Crystal data

[Ag_3_(C_9_H_6_N_3_O_3_S)_3_(H_2_O)_2_]
*M* *_r_* = 1068.36
Orthorhombic, 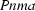

*a* = 19.0738 (16) Å
*b* = 19.9598 (17) Å
*c* = 8.6372 (7) Å
*V* = 3288.3 (5) Å^3^
*Z* = 4
Mo *K*α radiation
μ = 2.04 mm^−1^
*T* = 291 K
0.30 × 0.24 × 0.22 mm

#### Data collection

Bruker SMART CCD area-detector diffractometer
Absorption correction: multi-scan (*SADABS*; Bruker, 2000 ▶) *T*_min_ = 0.56, *T*_max_ = 0.64
16896 measured reflections
3327 independent reflections
2555 reflections with *I* > 2σ(*I*)
*R*_int_ = 0.062

#### Refinement

*R*[*F* ^2^ > 2σ(*F* ^2^)] = 0.052
*wR*(*F* ^2^) = 0.119
*S* = 1.07
3327 reflections
274 parameters
H-atom parameters constrained
Δρ_max_ = 0.80 e Å^−3^
Δρ_min_ = −1.25 e Å^−3^

### 

Data collection: *SMART* (Bruker, 2000 ▶); cell refinement: *SAINT* (Bruker, 2000 ▶); data reduction: *SAINT*; program(s) used to solve structure: *SHELXTL* (Sheldrick, 2008 ▶); program(s) used to refine structure: *SHELXTL*; molecular graphics: *SHELXTL*; software used to prepare material for publication: *SHELXTL*.

## Supplementary Material

Crystal structure: contains datablock(s) I, global. DOI: 10.1107/S1600536811026626/cv5105sup1.cif

Structure factors: contains datablock(s) I. DOI: 10.1107/S1600536811026626/cv5105Isup2.hkl

Additional supplementary materials:  crystallographic information; 3D view; checkCIF report

## Figures and Tables

**Table 1:** Hydrogen-bond geometry (Å, °)

*D*—H⋯*A*	*D*—H	H⋯*A*	*D*⋯*A*	*D*—H⋯*A*
O4—H4*C*⋯O2^i^	0.96	1.83	2.784 (6)	171

Symmetry code: (i) 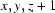.

**Fig. 1.**
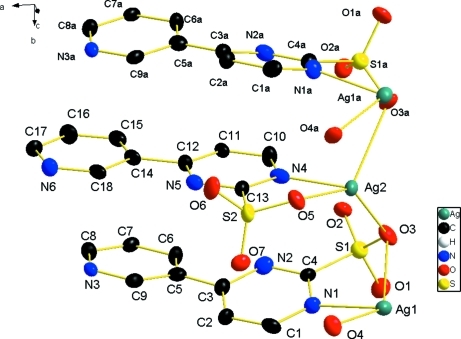
A portion of the polymeric structure of (I) showing the atomic numbering and 30% probability displacement ellipsoids [symmetry code: (a) x, 0.5 - y, z]. For the disordered sulfonato group with the atoms O5, O6 and O7 only one orientation is shown.

## supplementary crystallographic information

### Comment

Remarkable attention has been paid to the rational design and assembly of new coordination polymers with thioethers in recent years (Dong *et al.*, 2009; Fang *et al.*, 2010; Zhu, 2010). Herein we present the title compound, (I) - the newly synthesized compound derived from 4-(2-pyridinyl)pyrimidine-2-thiol.

The molecular structure of title compound with the atom-numbering scheme is shown in Fig. 1. It crystallizes in the centrosymmetric space group *\'-P 2ac 2n'*. Ag1 is tetracoordinated and Ag2 is pentacoordinate. Ag1 is coordinated by two N atom from two *L* ligands and one coordinated water molecule as well as one O atom from the SO_3_ group. The two N atoms from different ligands are in a slightly distorted linear geometry with an N—Ag—N bond angle of 160.8°. Ag2 is bound by two different N atoms located from two ligands, and thee O donors from the three different SO3 group.

In (I) (Fig. 1), each ligand *L* (*L* = 4-(2-pyridyl)-pyrimidine-2-sulfonato) exhibits a chelating-bridging tridentate mode. The molecules are linked into three-decked polymeric zigzag chains propagated in [100]. On the middle deck, which is situated on a mirror plane, the Ag centre is five-coordinated by three O atoms from three ligands *L*, one of which lies on a mirror plane with the disordered sulfonato group over two orientations in a 1:1 ratio, and two N atoms from two ligands *L*, which lie on the same mirror plane. On the upper and lower decks, each Ag centre is four-coordinated by aqua ligand, one O and two N atoms from two ligands *L* with the pyridyl and pyrimidine rings twisted at 19.8 (2)°. In the polymeric chain, there are π–π interactions between six-membered rings of ligands *L* from different decks (Table 1).

In the crystal structure, intermolecular O—H···O hydrogen bonds l(Table 2) ink further these three-decked chains into layers parallel to *ac* plane.

### Experimental

All solvents and chemicals were of analytical grade and were used without further purification. The title compound was prepared by similar procedure reported in the literature (Dong *et al.*, 2009; Fang *et al.*, 2010; Zhu, 2010). To a suspension of Na*L*_2_ (26.0 mg, 0.1 mmol) in water (5 ml) in a tube, a solution of AgNO_3_ (8.5 mg, 0.05 mmol) in acetonitrile (5 ml) was very slowly dropped. Crystal products formed after two weeks standing in a dark. Single crystals suitable for X-ray diffraction were grown from methanol solution by slow evaporation in air at room temperature.

### Refinement

All hydrogen atoms were geometrically positioned (C—H 0.93 Å; O—H 0.96 Å) and refined as riding, with *U*_iso_(H)=1.2–1.5 *U*_eq_ of the parent atom.

### Crystal data

**Table:**

[Ag_3_(C_9_H_6_N_3_O_3_S)_3_(H_2_O)_2_]	*F*(000) = 2096
*M**_r_* = 1068.36	*D*_x_ = 2.158 Mg m^−^^3^
Orthorhombic, *P**n**m**a*	Mo *K*α radiation, λ = 0.71073 Å
Hall symbol: -P 2ac 2n	Cell parameters from 3327 reflections
*a* = 19.0738 (16) Å	θ = 2.1–26.0°
*b* = 19.9598 (17) Å	µ = 2.04 mm^−^^1^
*c* = 8.6372 (7) Å	*T* = 291 K
*V* = 3288.3 (5) Å^3^	Block, colourless
*Z* = 4	0.30 × 0.24 × 0.22 mm

### Data collection

**Table:**

Bruker SMART CCD area-detector diffractometer	3327 independent reflections
Radiation source: fine-focus sealed tube	2555 reflections with *I* > 2σ(*I*)
graphite	*R*_int_ = 0.062
φ and ω scan	θ_max_ = 26.0°, θ_min_ = 2.1°
Absorption correction: multi-scan (*SADABS*; Bruker, 2000)	*h* = −15→23
*T*_min_ = 0.56, *T*_max_ = 0.64	*k* = −24→24
16896 measured reflections	*l* = −10→10

### Refinement

**Table:**

Refinement on *F*^2^	Primary atom site location: structure-invariant direct methods
Least-squares matrix: full	Secondary atom site location: difference Fourier map
*R*[*F*^2^ > 2σ(*F*^2^)] = 0.052	Hydrogen site location: inferred from neighbouring sites
*wR*(*F*^2^) = 0.119	H-atom parameters constrained
*S* = 1.07	*w* = 1/[σ^2^(*F*_o_^2^) + (0.06*P*)^2^ + 1.55*P*] where *P* = (*F*_o_^2^ + 2*F*_c_^2^)/3
3327 reflections	(Δ/σ)_max_ < 0.001
274 parameters	Δρ_max_ = 0.80 e Å^−^^3^
0 restraints	Δρ_min_ = −1.25 e Å^−^^3^

### Special details

**Table:**

Experimental. The structure was solved by direct methods (Bruker, 2000) and successive difference Fourier syntheses.
Geometry. All e.s.d.'s (except the e.s.d. in the dihedral angle between two l.s. planes) are estimated using the full covariance matrix. The cell e.s.d.'s are taken into account individually in the estimation of e.s.d.'s in distances, angles and torsion angles; correlations between e.s.d.'s in cell parameters are only used when they are defined by crystal symmetry. An approximate (isotropic) treatment of cell e.s.d.'s is used for estimating e.s.d.'s involving l.s. planes.
Refinement. Refinement of *F*^2^ against ALL reflections. The weighted *R*-factor *wR* and goodness of fit *S* are based on *F*^2^, conventional *R*-factors *R* are based on *F*, with *F* set to zero for negative *F*^2^. The threshold expression of *F*^2^ > σ(*F*^2^) is used only for calculating *R*-factors(gt) *etc*. and is not relevant to the choice of reflections for refinement. *R*-factors based on *F*^2^ are statistically about twice as large as those based on *F*, and *R*- factors based on ALL data will be even larger.

### Fractional atomic coordinates and isotropic or equivalent isotropic displacement parameters (Å^2^)

**Table:**

	*x*	*y*	*z*	*U*_iso_*/*U*_eq_	Occ. (<1)
Ag1	0.13631 (3)	0.41025 (2)	0.94740 (6)	0.03754 (16)	
Ag2	0.17483 (3)	0.2500	0.82162 (8)	0.03481 (19)	
C1	0.2943 (4)	0.4436 (3)	0.8566 (7)	0.0408 (15)	
H1	0.2986	0.4544	0.9609	0.049*	
C2	0.3536 (4)	0.4436 (3)	0.7610 (7)	0.0374 (14)	
H2	0.3972	0.4546	0.8018	0.045*	
C3	0.3474 (4)	0.4272 (3)	0.6065 (8)	0.0381 (15)	
C4	0.2242 (3)	0.4108 (3)	0.6403 (7)	0.0335 (13)	
C5	0.4053 (4)	0.4241 (3)	0.4987 (8)	0.0384 (14)	
C6	0.3956 (3)	0.4261 (3)	0.3385 (8)	0.0396 (15)	
H6	0.3507	0.4293	0.2971	0.047*	
C7	0.4540 (3)	0.4234 (3)	0.2411 (7)	0.0326 (13)	
H7	0.4479	0.4252	0.1344	0.039*	
C8	0.5201 (3)	0.4181 (3)	0.3014 (7)	0.0392 (14)	
H8	0.5587	0.4162	0.2357	0.047*	
C9	0.4730 (3)	0.4183 (3)	0.5592 (7)	0.0351 (14)	
H9	0.4795	0.4163	0.6658	0.042*	
C10	0.2727 (5)	0.2500	0.5262 (11)	0.044 (2)	
H10	0.2316	0.2500	0.4683	0.053*	
C11	0.3369 (4)	0.2500	0.4532 (10)	0.0311 (18)	
H11	0.3390	0.2500	0.3456	0.037*	
C12	0.3972 (4)	0.2500	0.5375 (10)	0.0330 (19)	
C13	0.3317 (5)	0.2500	0.7715 (10)	0.0348 (19)	
C14	0.4694 (4)	0.2500	0.4665 (9)	0.0329 (19)	
C15	0.4792 (5)	0.2500	0.3072 (11)	0.041 (2)	
H15	0.4407	0.2500	0.2410	0.049*	
C16	0.5466 (5)	0.2500	0.2474 (11)	0.044 (2)	
H16	0.5530	0.2500	0.1406	0.053*	
C17	0.6043 (5)	0.2500	0.3432 (9)	0.0337 (19)	
H17	0.6493	0.2500	0.3022	0.040*	
C18	0.5278 (5)	0.2500	0.5643 (11)	0.0345 (19)	
H18	0.5216	0.2500	0.6712	0.041*	
N1	0.2295 (3)	0.4278 (3)	0.7962 (6)	0.0409 (13)	
N2	0.2817 (3)	0.4101 (3)	0.5462 (6)	0.0421 (13)	
N3	0.5297 (3)	0.4155 (2)	0.4619 (6)	0.0323 (11)	
N4	0.2698 (4)	0.2500	0.6895 (8)	0.0366 (17)	
N5	0.3958 (4)	0.2500	0.6979 (9)	0.0358 (17)	
N6	0.5937 (4)	0.2500	0.5036 (9)	0.0394 (18)	
O1	0.1096 (2)	0.4554 (2)	0.5436 (5)	0.0450 (12)	
O2	0.1566 (3)	0.3564 (2)	0.4227 (5)	0.0458 (11)	
O3	0.1103 (2)	0.3505 (2)	0.6811 (5)	0.0432 (11)	
O4	0.2270 (2)	0.3934 (2)	1.1536 (5)	0.0415 (11)	
H4B	0.2560	0.3560	1.1266	0.062*	
H4C	0.2041	0.3848	1.2506	0.062*	
O5	0.2702 (5)	0.2734 (5)	1.0142 (10)	0.045 (2)	0.50
O6	0.3935 (6)	0.2966 (6)	1.0009 (12)	0.048 (3)	0.50
O7	0.3524 (6)	0.1854 (5)	1.0227 (10)	0.039 (2)	0.50
S1	0.14301 (9)	0.39214 (9)	0.5640 (2)	0.0420 (4)	
S2	0.33833 (12)	0.2500	0.9713 (3)	0.0387 (5)	

### Atomic displacement parameters (Å^2^)

**Table:**

	*U*^11^	*U*^22^	*U*^33^	*U*^12^	*U*^13^	*U*^23^
Ag1	0.0395 (3)	0.0334 (3)	0.0398 (3)	−0.0030 (2)	0.0070 (2)	−0.0051 (2)
Ag2	0.0272 (3)	0.0338 (3)	0.0434 (4)	0.000	0.0094 (3)	0.000
C1	0.048 (4)	0.038 (4)	0.036 (3)	−0.009 (3)	−0.012 (3)	−0.004 (3)
C2	0.037 (4)	0.042 (3)	0.033 (3)	0.000 (3)	0.010 (3)	−0.005 (3)
C3	0.036 (4)	0.037 (3)	0.041 (3)	0.004 (3)	0.007 (3)	0.004 (3)
C4	0.037 (3)	0.029 (3)	0.034 (3)	−0.001 (3)	0.007 (3)	−0.007 (2)
C5	0.046 (4)	0.029 (3)	0.040 (3)	0.001 (3)	−0.004 (3)	−0.005 (3)
C6	0.029 (3)	0.051 (4)	0.038 (3)	0.003 (3)	0.006 (3)	0.002 (3)
C7	0.043 (4)	0.029 (3)	0.026 (3)	0.002 (3)	0.011 (3)	0.003 (2)
C8	0.033 (3)	0.047 (4)	0.037 (3)	−0.004 (3)	0.000 (3)	0.002 (3)
C9	0.047 (4)	0.027 (3)	0.032 (3)	−0.003 (3)	0.010 (3)	0.001 (2)
C10	0.032 (5)	0.063 (7)	0.037 (5)	0.000	0.002 (4)	0.000
C11	0.026 (4)	0.030 (4)	0.037 (5)	0.000	−0.010 (3)	0.000
C12	0.027 (5)	0.040 (5)	0.033 (5)	0.000	−0.004 (3)	0.000
C13	0.031 (5)	0.034 (4)	0.039 (5)	0.000	0.008 (4)	0.000
C14	0.031 (5)	0.036 (5)	0.032 (4)	0.000	−0.019 (3)	0.000
C15	0.038 (5)	0.035 (5)	0.050 (6)	0.000	−0.015 (4)	0.000
C16	0.040 (6)	0.058 (6)	0.035 (5)	0.000	−0.013 (4)	0.000
C17	0.040 (5)	0.040 (5)	0.021 (4)	0.000	0.006 (3)	0.000
C18	0.034 (5)	0.030 (4)	0.040 (5)	0.000	−0.019 (4)	0.000
N1	0.039 (3)	0.048 (3)	0.036 (3)	0.001 (2)	0.001 (2)	−0.010 (2)
N2	0.048 (3)	0.045 (3)	0.034 (3)	0.008 (3)	−0.007 (2)	0.003 (2)
N3	0.025 (3)	0.028 (3)	0.044 (3)	−0.002 (2)	−0.004 (2)	−0.002 (2)
N4	0.047 (5)	0.034 (4)	0.029 (4)	0.000	−0.022 (3)	0.000
N5	0.036 (4)	0.034 (4)	0.038 (4)	0.000	−0.011 (3)	0.000
N6	0.035 (4)	0.049 (5)	0.035 (4)	0.000	−0.006 (3)	0.000
O1	0.048 (3)	0.050 (3)	0.038 (2)	0.016 (2)	−0.010 (2)	0.011 (2)
O2	0.046 (3)	0.042 (3)	0.049 (3)	−0.007 (2)	0.001 (2)	−0.014 (2)
O3	0.045 (3)	0.049 (3)	0.036 (2)	−0.019 (2)	−0.008 (2)	0.017 (2)
O4	0.043 (3)	0.053 (3)	0.029 (2)	−0.005 (2)	−0.0050 (19)	0.0173 (19)
O5	0.039 (5)	0.051 (6)	0.045 (5)	0.013 (4)	−0.004 (4)	−0.009 (4)
O6	0.033 (6)	0.069 (8)	0.042 (6)	−0.016 (5)	−0.009 (5)	−0.010 (5)
O7	0.054 (7)	0.039 (5)	0.025 (5)	−0.004 (5)	0.004 (4)	−0.003 (4)
S1	0.0421 (10)	0.0402 (9)	0.0438 (9)	−0.0010 (7)	−0.0005 (7)	0.0014 (7)
S2	0.0260 (11)	0.0504 (14)	0.0396 (12)	0.000	0.0029 (9)	0.000

### Geometric parameters (Å, °)

**Table:**

Ag1—N3^i^	2.182 (5)	C12—C14	1.508 (13)
Ag1—N1	2.234 (6)	C13—N5	1.377 (12)
Ag1—O4	2.505 (4)	C13—N4	1.377 (11)
Ag2—N4	2.140 (8)	C13—S2	1.730 (9)
Ag2—N6^i^	2.162 (8)	C14—C15	1.389 (13)
Ag2—O5	2.508 (9)	C14—C18	1.397 (11)
Ag2—O5^ii^	2.508 (9)	C15—C16	1.386 (14)
C1—N1	1.378 (8)	C15—H15	0.9300
C1—C2	1.400 (9)	C16—C17	1.376 (13)
C1—H1	0.9300	C16—H16	0.9300
C2—C3	1.380 (9)	C17—N6	1.400 (11)
C2—H2	0.9300	C17—H17	0.9300
C3—N2	1.401 (9)	C18—N6	1.363 (12)
C3—C5	1.445 (9)	C18—H18	0.9300
C4—N2	1.364 (8)	N3—Ag1^iii^	2.182 (5)
C4—N1	1.392 (8)	N6—Ag2^iii^	2.162 (8)
C4—S1	1.724 (6)	O1—S1	1.424 (5)
C5—C6	1.396 (9)	O2—S1	1.438 (5)
C5—C9	1.397 (9)	O3—S1	1.449 (4)
C6—C7	1.397 (9)	O4—H4B	0.9600
C6—H6	0.9300	O4—H4C	0.9600
C7—C8	1.368 (9)	O5—O5^ii^	0.934 (18)
C7—H7	0.9300	O5—S2	1.431 (9)
C8—N3	1.399 (8)	O5—O7^ii^	1.773 (14)
C8—H8	0.9300	O6—O7^ii^	0.882 (12)
C9—N3	1.371 (8)	O6—S2	1.428 (10)
C9—H9	0.9300	O7—O6^ii^	0.882 (12)
C10—C11	1.377 (12)	O7—S2	1.389 (10)
C10—N4	1.412 (11)	O7—O5^ii^	1.773 (14)
C10—H10	0.9300	S2—O7^ii^	1.389 (10)
C11—C12	1.362 (11)	S2—O6^ii^	1.428 (10)
C11—H11	0.9300	S2—O5^ii^	1.431 (9)
C12—N5	1.386 (11)		
			
Cg1···Cg1^ii^	3.621 (7)	Cg2···Cg2^ii^	3.721 (3)
			
N3^i^—Ag1—N1	160.8 (2)	C16—C17—N6	118.7 (8)
N3^i^—Ag1—O4	113.25 (17)	C16—C17—H17	120.6
N1—Ag1—O4	83.53 (17)	N6—C17—H17	120.6
N4—Ag2—N6^i^	167.9 (3)	N6—C18—C14	120.1 (8)
N4—Ag2—O5	74.9 (3)	N6—C18—H18	119.9
N6^i^—Ag2—O5	93.2 (3)	C14—C18—H18	119.9
N4—Ag2—O5^ii^	74.9 (3)	C1—N1—C4	119.2 (5)
N6^i^—Ag2—O5^ii^	93.2 (3)	C1—N1—Ag1	121.9 (4)
O5—Ag2—O5^ii^	21.5 (4)	C4—N1—Ag1	118.0 (4)
N1—C1—C2	120.0 (5)	C4—N2—C3	119.7 (5)
N1—C1—H1	120.0	C9—N3—C8	120.2 (5)
C2—C1—H1	120.0	C9—N3—Ag1^iii^	121.2 (4)
C3—C2—C1	120.1 (6)	C8—N3—Ag1^iii^	118.6 (4)
C3—C2—H2	119.9	C13—N4—C10	118.7 (8)
C1—C2—H2	119.9	C13—N4—Ag2	116.8 (5)
C2—C3—N2	119.6 (6)	C10—N4—Ag2	124.5 (6)
C2—C3—C5	124.6 (6)	C13—N5—C12	118.7 (7)
N2—C3—C5	115.7 (6)	C18—N6—C17	120.9 (8)
N2—C4—N1	121.4 (6)	C18—N6—Ag2^iii^	113.0 (6)
N2—C4—S1	119.5 (4)	C17—N6—Ag2^iii^	126.0 (6)
N1—C4—S1	119.2 (5)	Ag1—O4—H4B	109.3
C6—C5—C9	119.7 (6)	Ag1—O4—H4C	109.4
C6—C5—C3	122.4 (6)	H4B—O4—H4C	109.5
C9—C5—C3	117.9 (6)	O5^ii^—O5—S2	70.9 (4)
C5—C6—C7	119.3 (6)	O5^ii^—O5—O7^ii^	117.6 (4)
C5—C6—H6	120.3	S2—O5—O7^ii^	50.0 (4)
C7—C6—H6	120.3	O5^ii^—O5—Ag2	79.3 (2)
C8—C7—C6	120.6 (6)	S2—O5—Ag2	115.2 (5)
C8—C7—H7	119.7	O7^ii^—O5—Ag2	139.1 (6)
C6—C7—H7	119.7	O7^ii^—O6—S2	69.4 (9)
C7—C8—N3	120.0 (6)	O6^ii^—O7—S2	74.2 (10)
C7—C8—H8	120.0	O6^ii^—O7—O5^ii^	126.1 (12)
N3—C8—H8	120.0	S2—O7—O5^ii^	52.1 (4)
N3—C9—C5	120.2 (6)	O1—S1—O2	114.5 (3)
N3—C9—H9	119.9	O1—S1—O3	113.7 (3)
C5—C9—H9	119.9	O2—S1—O3	112.7 (3)
C11—C10—N4	119.5 (9)	O1—S1—C4	104.9 (3)
C11—C10—H10	120.2	O2—S1—C4	105.7 (3)
N4—C10—H10	120.2	O3—S1—C4	104.1 (3)
C12—C11—C10	120.4 (8)	O7^ii^—S2—O7	136.2 (8)
C12—C11—H11	119.8	O7—S2—O6	113.9 (8)
C10—C11—H11	119.8	O7^ii^—S2—O6^ii^	113.9 (8)
C11—C12—N5	121.2 (8)	O6—S2—O6^ii^	81.3 (10)
C11—C12—C14	123.7 (8)	O7^ii^—S2—O5^ii^	113.3 (6)
N5—C12—C14	115.1 (7)	O7—S2—O5^ii^	77.9 (6)
N5—C13—N4	121.5 (8)	O6—S2—O5^ii^	146.8 (6)
N5—C13—S2	113.3 (7)	O6^ii^—S2—O5^ii^	114.3 (6)
N4—C13—S2	125.1 (7)	O7^ii^—S2—O5	77.9 (6)
C15—C14—C18	119.5 (9)	O7—S2—O5	113.3 (6)
C15—C14—C12	121.7 (7)	O6—S2—O5	114.3 (6)
C18—C14—C12	118.8 (8)	O6^ii^—S2—O5	146.8 (6)
C16—C15—C14	119.6 (8)	O7^ii^—S2—C13	109.4 (4)
C16—C15—H15	120.2	O7—S2—C13	109.4 (4)
C14—C15—H15	120.2	O6—S2—C13	103.4 (5)
C17—C16—C15	121.1 (9)	O6^ii^—S2—C13	103.4 (5)
C17—C16—H16	119.4	O5^ii^—S2—C13	101.1 (5)
C15—C16—H16	119.4	O5—S2—C13	101.1 (5)

Symmetry codes: (i) *x*−1/2, *y*, −*z*+3/2; (ii) *x*, −*y*+1/2, *z*; (iii) *x*+1/2, *y*, −*z*+3/2.

### Hydrogen-bond geometry (Å, °)

**Table:**

*D*—H···*A*	*D*—H	H···*A*	*D*···*A*	*D*—H···*A*
O4—H4C···O2^iv^	0.96	1.83	2.784 (6)	171

Symmetry codes: (iv) *x*, *y*, *z*+1.
